# Overexpression of miR-101 promotes TRAIL-induced mitochondrial apoptosis in papillary thyroid carcinoma by targeting c-met and MCL-1

**DOI:** 10.18632/oncotarget.21215

**Published:** 2017-09-23

**Authors:** Jie Zhu, Zhenjie Li

**Affiliations:** ^1^ Department of Endocrinology, Linyi People’s Hospital, Linyi, China, 276000

**Keywords:** papillary thyroid carcinoma, miR-101, TRAIL, c-met, MCL-1

## Abstract

Tumor necrosis factor-related apoptosis inducing ligand (TRAIL) induces apoptosis in malignant cells, but not in normal cells. As papillary thyroid carcinoma cells broadly expressed TRAIL receptors (death receptor 4 and death receptor 5) on their surface, TRAIL is considered as a promising drug for treatment of papillary thyroid carcinoma. However, resistance to TRAIL still be a big obstacle to achieve a satisfactory effect for cancer therapy. Here, we found that overexpression of miR-101 was able to sensitize papillary thyroid carcinoma cells to TRAIL treatment *in vitro* and *in vivo*. Mechanically, we found that genes of c-met and MCL-1 were the targets of miR-101. Overexpression of miR-101 in TPC-1 significantly decreased the cellular protein levels of c-met and MCL-1, and thus inhibiting the PI3K/AKT pathway and reducing the resistance to TRAIL-induced mitochondrial apoptosis. Enforced expression of either c-met or MCL-1 could partially inhibit the miR-101 promoted apoptosis in TRAIL-treated TPC-1 cells. These results indicated that miR-101-c-met/MCL-1 axis determined the sensitivity of TRAIL to thyroid cancer in some extent. Combination with TRAIL and miR-101 may represent a novel approach to kill papillary thyroid carcinoma cells efficiently.

## INTRODUCTION

Papillary thyroid carcinoma (PTC) represents as the most common malignancy of the endocrine system, which is accounting for about 90% of the whole thyroid carcinoma cases [1, 2]. Although prognosis for patients with PTC is often favorable after surgical resection, about 10-15% of PTC tumors recur and occur distant metastasis [3, 4]. For these patients, systematic treatment is required. Unfortunately, PTC cells usually exhibited low sensitivity to chemo- or radio- therapy [5, 6].

Tumor necrosis factor-related apoptosis inducing ligand (TRAIL) binds to death receptor 4 (DR4) and death receptor 5 (DR5) which exists on cell surface, and then triggers caspase-8-dependent extrinsic and mitochondrial apoptosis [7, 8]. Selectively, TRAIL induces apoptosis in malignant cells, but not in normal cells. Furthermore, as papillary thyroid carcinoma cells broadly expressed DR4 and DR5 on their surface, TRAIL is considered as a promising drug for treatment of PTC [9, 10, 11]. However, some cancer cells develop mechanisms against TRAIL-mediated apoptosis [12, 13]. It’s urgent to identify the mechanisms and sensitize PTC cells to TRAIL-mediated apoptosis to achieve a satisfactory effect for cancer therapy.

MicroRNAs (miRNAs) are a class of endogenous and non-coding RNA oligonucleotides with 19-25 nucleotides. MiRNAs act as negative regulators of gene expression through binding to the complementary sequences of the 3’untranslated region (3’ UTR) of their target genes [14, 15]. It has been reported that about 60% of human genes are regulated by miRNAs. Furthermore, over 50% of these genes participate in cancer proliferation, metastasis and apoptosis [16, 17]. It is clear that miRNAs play important roles in cancer biological processes. Recently, studies demonstrate that aberrant expression of miRNAs in cancers is responsible for resistance to various anti-tumor drugs including TRAIL [18, 19, 20]. In the present study, we found that expression of miR-101 was decreased in PTC cells. Overexpression of miR-101 was able to sensitize PTC cells to TRAIL treatment *in vitro* and *in vivo* by targeting c-met and MCL-1.

## RESULTS

### Overexpression of miR-101 sensitizes PTC cells to TRAIL-induced cell death

**Figure 1:**
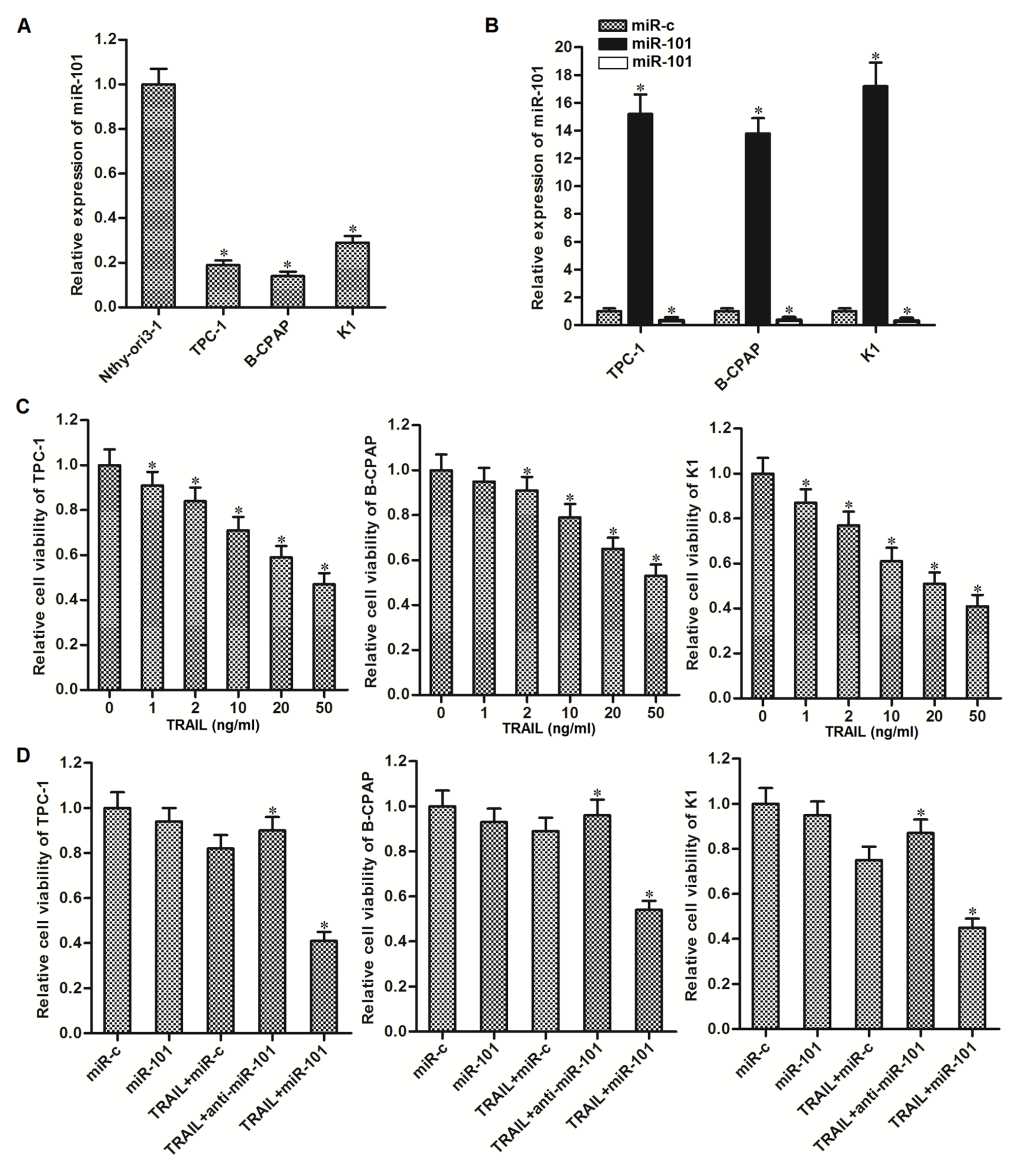
Overexpression of miR-101 sensitizes PTC cells to TRAIL-induced cell death **(A)** QRT-PCR analysis was performed to detect the expression level of miR-101 in human thyroid epithelial cell line Nthy-ori3-1 and TPC-1, BCPAP and K1 PTC cell lines. ^*^P<0.05 *vs.* Nthy-ori3-1 cells. **(B)** Effect of miR-101 mimics or inhibitors transfection on changing cellular level of miR-101 was evaluated by qRT-PCR analysis. ^*^P<0.05 *vs.* miR-c group. **(C)** TPC-1, BCPAP and K1 cells were treated with different concentrations of TRAIL for 48 h. MTT assays were performed to evaluate the cell viability of them. ^*^P<0.05 *vs.* control group. **(D)** TPC-1, BCPAP and K1 cells were transfected with miR-101 mimics or inhibitors (50 pmol/ml) before they were treated with TRAIL (2 ng/ml). MTT assays were performed to evaluate the cell viability of them. ^*^P<0.05 *vs.* TRAIL+miR-c group.

To explore the potential role of miR-101 in PTC, we first detected the expression of miR-101 in human thyroid epithelial cell line Nthy-ori3-1 and PTC cell lines (TPC-1, BCPAP and K1). We observed that expression level of miR-101 in PTC cells was significantly lower than that in Nthy-ori3-1 cells (Figure 1A). It suggested that aberrant expression of miR-101 was required in PTC. To study the potential benefit of miR-101 to sensitize PTC cells to TRAIL, we changed the cellular level of miR-101 by transfecting with miR-101 mimics or inhibitors (Figure 1B) before they were treated with TRAIL. In addition, the dose dependency of TRAIL to these PTC cancer cell lines was shown in Figure 1C. We chose the concentration of 2 ng/ml TRAIL which induced slight cell death of PTC cells for combination treatment with miR-101 mimics or inhibitors. Interestingly, we found that overexpression of miR-101 sensitizes PTC cells to TRAIL-induced cell death, whereas the anti-miR-101 reduced the cytotoxicity of TRAIL to these PTC cells (Figure 1C). Since sensitivity of TPC-1 to TRAIL single treatment was in the middle hierarchy, and the TRAIL-induced cell death in TPC-1 can be dramatically augmented, we performed our following experiments to examine the miR-101-induced changes in this PTC cell line.

### MiR-101 targets c-met and MCL-1 in PTC cells

**Figure 2:**
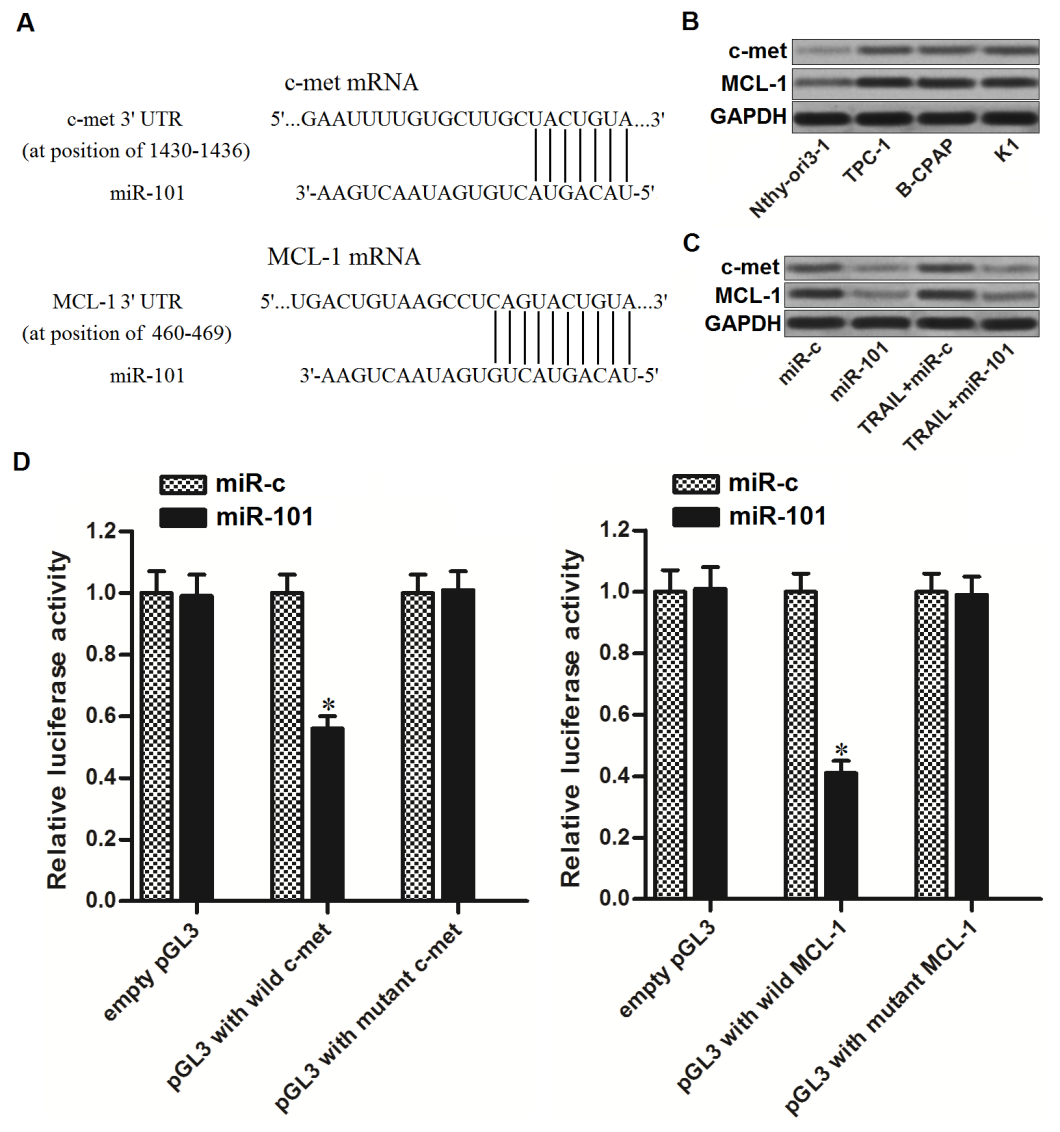
miR-101 targets c-met and MCL-1 in PTC cells **(A)** C-met and MCL-1 were predicted as the targets of miR-101 by the public databases of TargetScan, miRanda, and PicTar. **(B)** Protein levels of c-met and MCL-1 in human thyroid epithelial cell line Nthy-ori3-1 and TPC-1, BCPAP and K1 PTC cell lines were evaluated by western blot analysis. **(C)** Protein levels of c-met and MCL-1 in TPC-1 cells were evaluated after they were treated with miR-101 and TRAIL (2 ng/ml). **(D)** Dual-Luciferase Reporter Assay System was used to detect the luciferase activities in TPC-1 cells which were co-transfected with wildt/mutant 3’-UTR of c-met/MCL-1 and miR-101 mimics. ^*^P<0.05 *vs.* miR-c group.

To understand the mechanisms by which miR-101 sensitizes TRAIL-induced cell death in PTC, public databases (TargetScan, miRanda, and PicTar) were used to predict the targets of miR-101. Among the candidates, genes of c-met and MCL-1, which have been reported to be associated with TRAIL sensitivity to several cancers [21, 22], were commonly predicted by all of these databases and contain complementary sequences paired with miR-101 at the 3’ UTR (Figure 2A). Furthermore, contrary to decrease of miR-101 levels in PTC cells, expression of c-met and MCL-1 in PTC cells was obviously overexpressed compared to the human thyroid epithelial cell line Nthy-ori3-1 (Figure 2B). These results suggested the negative correlation between miR-101 and c-met/MCL-1. To confirm that miR-101 targeted c-met and MCL-1 in PTC cells, we detected the protein levels of c-met and MCL-1 in TPC-1 cells after they were treated with miR-101 and TRAIL. We found that overexpression of miR-101 significantly decreased the expression of both c-met and MCL-1 in TRAIL-treated PTC-1 cells (Figure 2C). Furthermore, results of luciferase reporter assays showed that miR-101 overexpression was able to shown to decrease the luciferase activity of pGL3 reporters with wild c-met or MCL-1 3’ UTR, while it failed to repress the pGL3 reporters with mutant c-met or MCL-1 3’ UTR in TPC-1 cells (Figure 2D). Taken together, we prove that miR-101 targeted c-met and MCL-1 in PTC cells.

### Expression of c-met and MCL-1 is associated with TRAIL sensitivity to PTC

**Figure 3:**
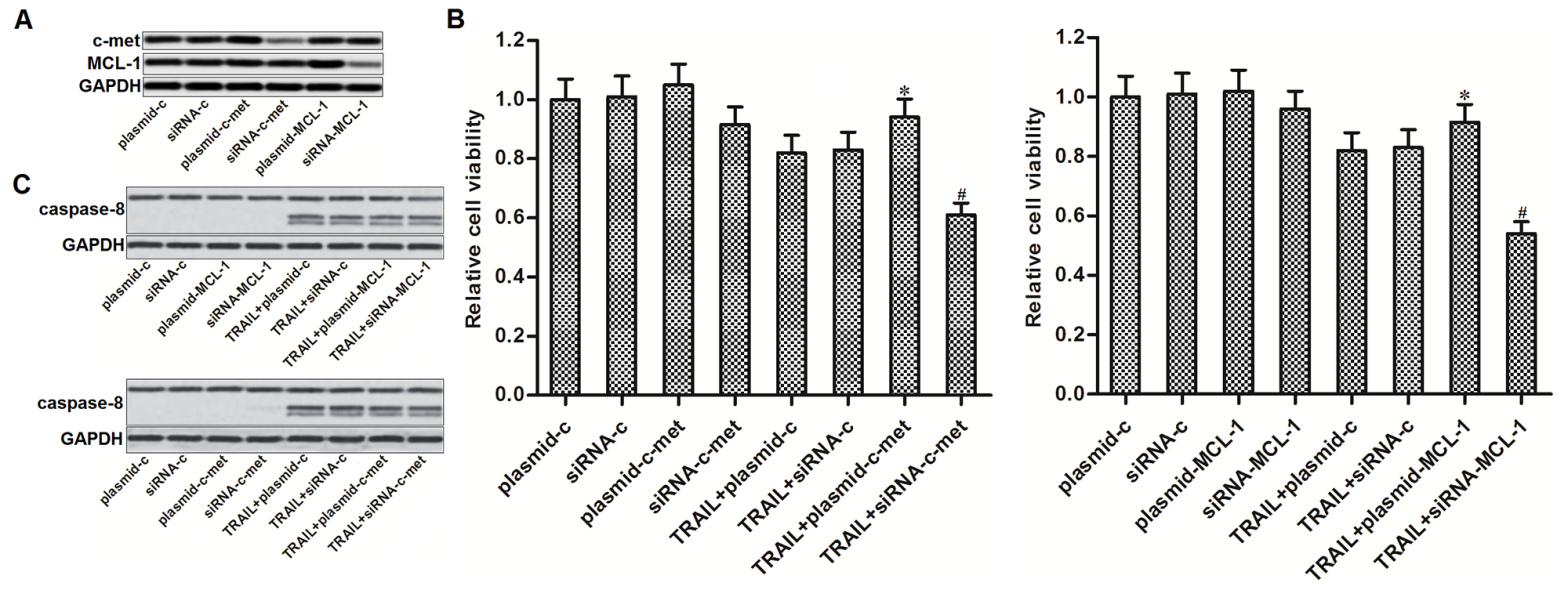
C-met and MCL-1 negatively regulated the TRAIL-induced cell death in PTC **(A)** Western blot analysis was performed to evaluate the transfection efficiency of c-met and MCL-1 plasmid and siRNA in TPC-1 cells. **(B)** MTT assays were performed to evaluate the gain- and loss-of-function of c-met and MCL-1 on TRAIL-induced cell death in TPC-1. ^*^P<0.05 *vs.* TRAIL+plasmid-c group. ^#^*P*<0.05 *vs.* TRAIL+siRNA-c group. **(C)** Western blot analysis was performed to evaluate the gain- and loss-of-function of c-met and MCL-1 on TRAIL-induced activation of caspase-8 in TPC-1 cells.

Since previous studies have demonstrated that c-met and MCL-1 regulate TRAIL sensitivity to several cancers [21, 22], we next investigated whether expression levels of c-met and MCL-1 were associated with TRAIL sensitivity to our PTC cell line TPC-1. We therefore performed gain- and loss-of-function experiments on c-met and MCL-1 by transfecting with eukaryotic expression vector and small interfering RNA (siRNA), respectively. Transfection efficiency of c-met and MCL-1 plasmid and siRNA was shown in Figure 3A. We found that overexpression of either c-met or MCL-1 partially reduced the TRAIL-induced cell death in TPC-1. On the contrary, knockdown of either c-met or MCL-1 significantly sensitized the TPC-1 cells to TRAIL-induced cell death (Figure 3B). However, gain- and loss-of-function of c-met and MCL-1 failed to change the TRAIL-induced activation of caspase-8 in TPC-1 (Figure 3C). These results suggested that c-met and MCL-1 negatively regulated the TRAIL-induced cell death in PTC through inhibiting the downstream of caspase-8 pathway.

### MiR-101 sensitizes TPC-1 cells to TRAIL via down-regulating the expression of c-met and MCL-1

**Figure 4:**
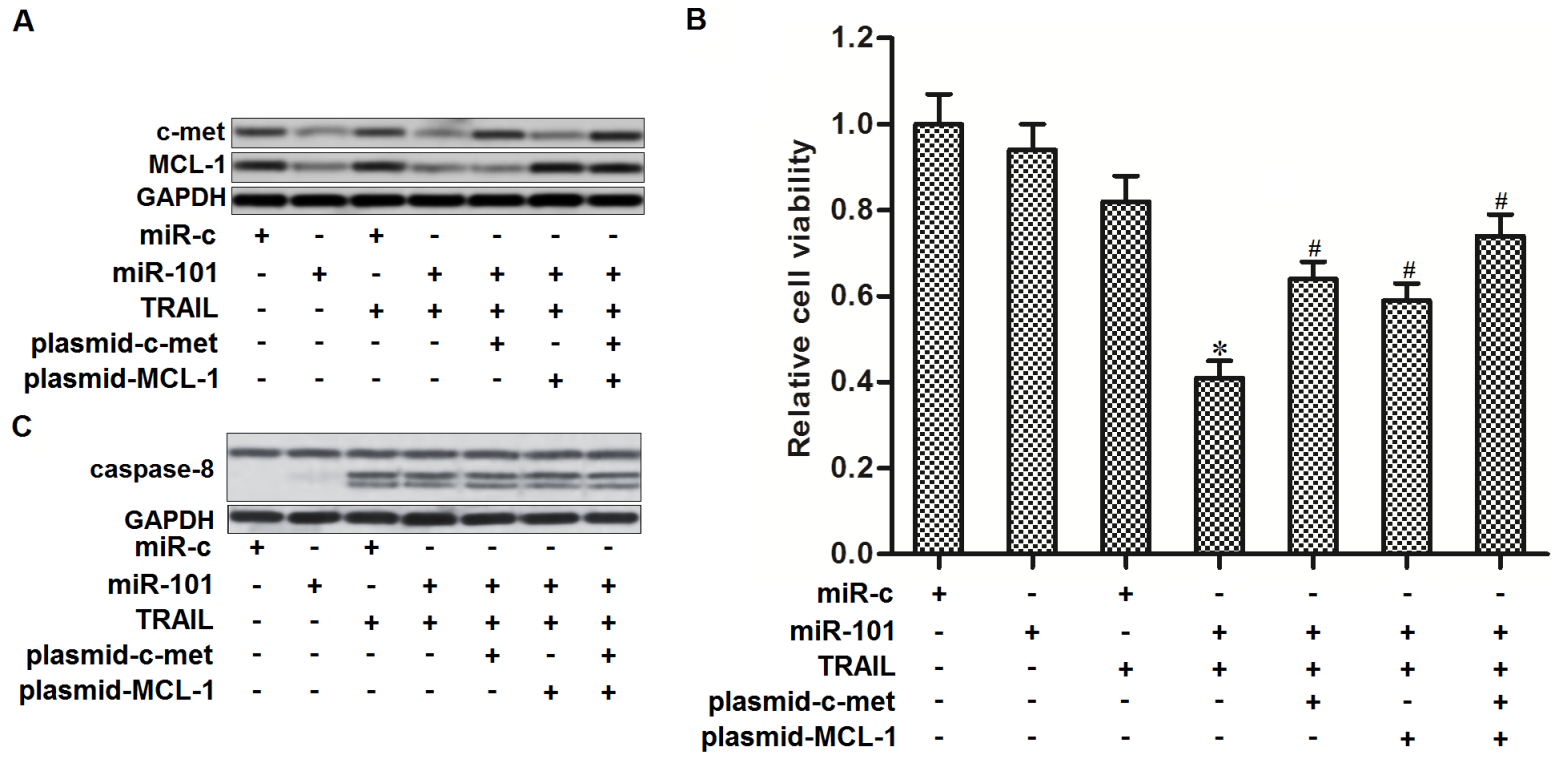
MiR-101 sensitizes TRAIL-induced cell death in TPC-1 by targeting c-met and MCL-1 **(A)** C-met and MCL-1 plasmid abolished miR-101-induced decrease of c-met and MCL-1 expression, respectively. **(B)** C-met and MCL-1 plasmid partially inhibited the effect of miR-101 on TRAIL-induced cell death in TPC-1 cells. ^*^P<0.05 *vs.* TRAIL+miR-c group. ^#^*P*<0.05 *vs.* TRAIL+miR-101 group. **(C)** Effect of miR-101, c-met and MCL-1 plasmid on TRAIL-induced activation of caspase-8 in TPC-1 cells.

As miR-101 targets c-met and MCL-1 in PTC, we next studied the role of c-met and MCL-1 in miR-101-promoted cell death in TPC-1 cells. We showed that transfection with c-met and MCL-1 plasmid abolished the effect of miR-101 on suppressing the expression of c-met and MCL-1, respectively (Figure 4A). Moreover, both c-met and MCL-1 plasmid partially inhibited the effect of miR-101 on TRAIL-induced cell death in TPC-1 cells (Figure 4B). In addition, overexpression of miR-101 failed to promote the TRAIL-induced activation of caspase-8 in TPC-1 cells (Figure 4C). We demonstrated that miR-101 promoted TRAIL-induced cell death in PTC by targeting c-met and MCL-1 which were the downstream regulators in caspase-8 pathway.

### Overexpression of miR-101 promotes TRAIL-induced mitochondrial apoptosis in PTC

**Figure 5:**
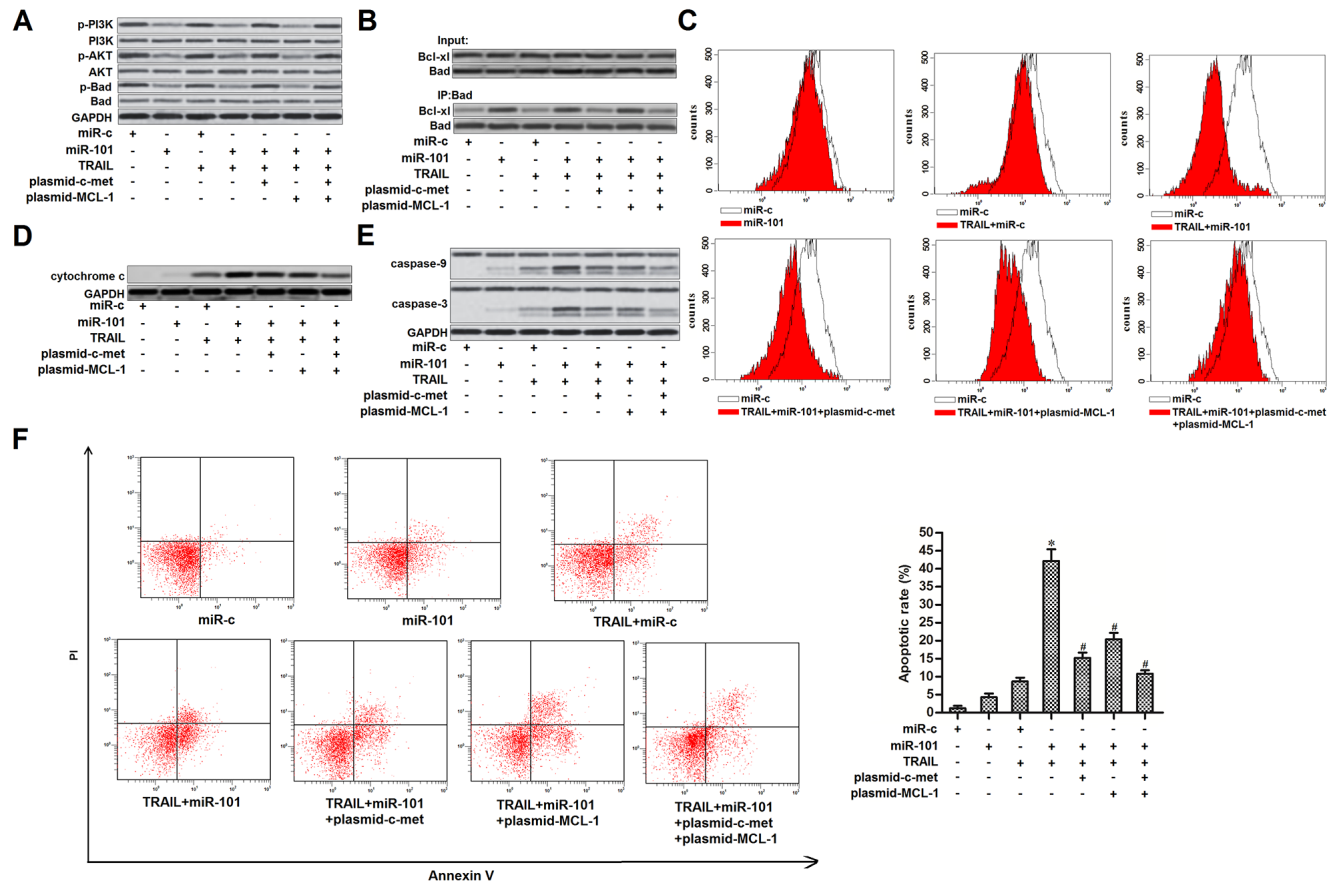
MiR-101 promoted TRAIL-induced mitochondrial apoptosis in TPC-1 **(A)** TPC-1 cells were transfected with miR-101, c-met and MCL-1 plasmid before they were treated with TRAIL (2 ng/ml). western blot analysis was performed to evaluate the phosphorylation of PI3K, AKT and Bad. **(B)** Co-immunoprecipitation was used to examine the effect of miR-101 on changing the interaction with Bad and MCL-1 in TPC-1 cells. **(C)** TPC-1 cells were transfected with miR-101, c-met and MCL-1 plasmid before they were treated with TRAIL (2 ng/ml). Flow cytometry analysis was performed to examine the mitochondrial membrane potential. **(D)** After removal of mitochondria, cytochrome c in cytoplasm was detected by western blot analysis. **(E)** After treatment with TRAIL (2 ng/ml), miR-101, c-met and MCL-1 plasmid, activation of caspase-9 and -3 was examined by using western blot analysis. **(F)** After treatment with TRAIL (2 ng/ml), miR-101, c-met and MCL-1 plasmid, apoptotic rate of TPC-1 was detected using flow cytometry. ^*^P<0.05 *vs.* TRAIL+miR-c group. ^#^*P*<0.05 *vs.* TRAIL+miR-101 group.

Results of western blot analysis showed that overexpression of miR-101 inhibited phosphorylation of PI3K and AKT in TPC-1 cells. As Bad is one of the substrates for AKT [23], we observed that introduction with miR-101 induced dephosphorylation of Bad. However, enforced expression of c-met was found to restore the phosphorylation of PI3K, AKT and its substrate of Bad in miR-101-overexpressed TPC-1 cells (Figure 5A). These results indicated that overexpression of miR-101 induced dephosphorylation of Bad through the c-met/PI3K/AKT pathway. Dephosphorylated Bad functions as a pro-apoptotic protein via interacting with Bcl-xl, and thus inactivating it which is a powerful anti-apoptotic protein [24]. As the dephosphorylated Bad was significantly increased in miR-101-overexpressed TPC-1 cells, our results showed that overexpression of miR-101 obviously enhanced the interaction with Bad and Bcl-xl (Figure 5B). Studies have proved that both Bcl-xl and MCL-1 functions as a suppressor in TRAIL-induced mitochondrial apoptosis [22, 25]. As miR-101 decreased the expression of MCL-1 and inactivated the Bcl-xl, we next investigate the role of miR-101 in TRAIL-induced mitochondrial apoptosis in TPC-1 cells. As shown in Figure 5C, overexpression of miR-101 dramatically enhanced the TRAIL-induced collapse of mitochondrial membrane potential (MMP). Furthermore, enforced expression of both c-met and MCL-1 partially inhibited the MMP collapse in TRAIL-treated TPC-1 cells. Due to the damage of mitochondria, the TRAIL- and miR-101-cotreated TPC-1 cells released more amount of cytochrome c from the mitochondria compared to the TPC-1 cells treated with TRAIL alone (Figure 5D). As cytochrome c in cytoplasm triggers caspase-9 via inducing Apaf-1/caspase-9 complex [20], we showed that overexpression of miR-101 significantly enhanced the TRAIL-induced activation of caspase-9 and -3 (Figure 5E) and occurrence of apoptosis (Figure 5F) in TPC-1 cells through inhibiting the expression of c-met and MCL-1. We concluded that overexpression of miR-101 was able to inhibit the expression of c-met and MCL-1, and thus promoting TRAIL-induced mitochondrial apoptosis in PTC.

### Overexpression of miR-101 enhances anti-tumor effect of TRAIL on PTC *in vivo*

**Figure 6:**
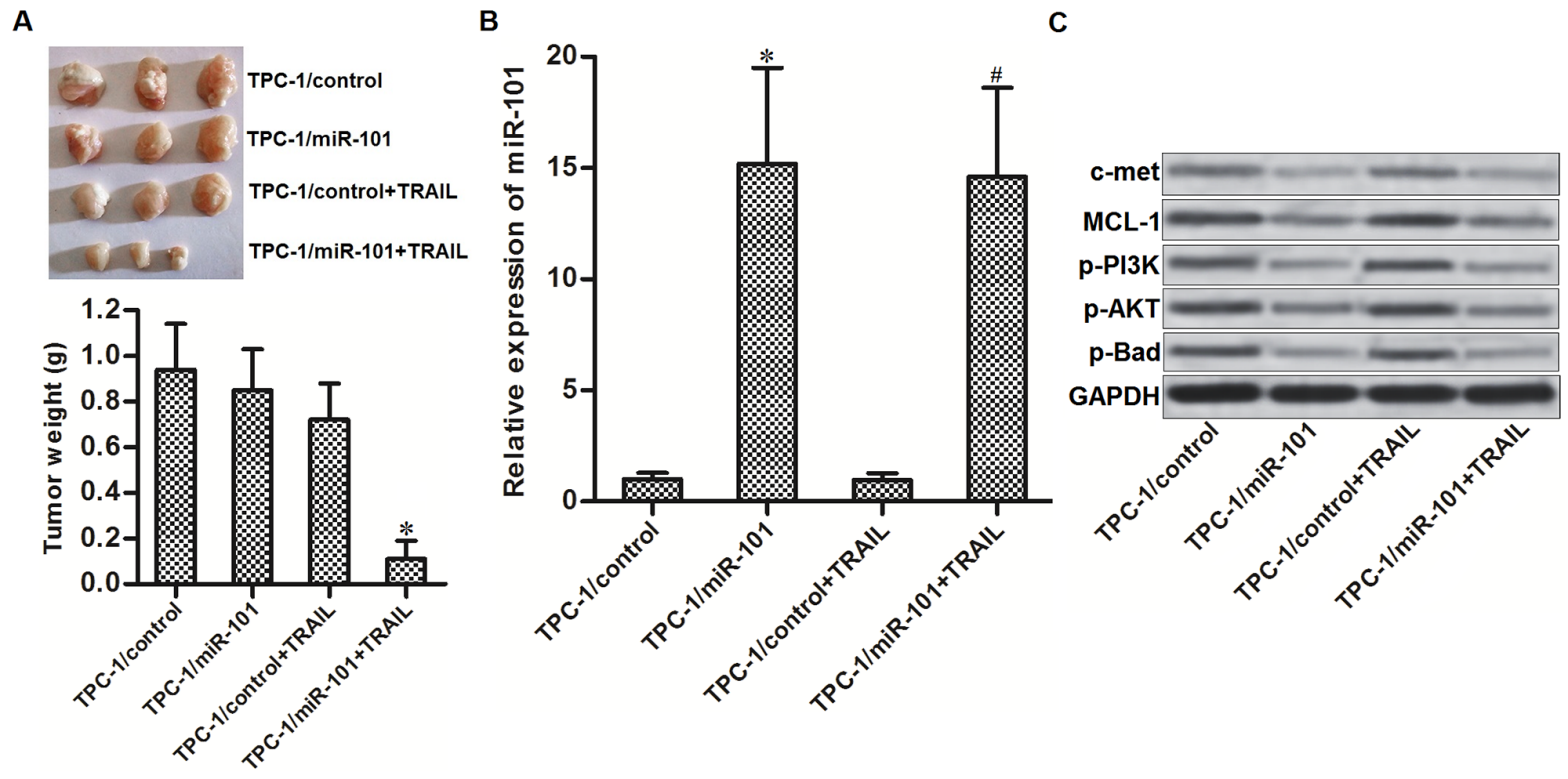
Overexpression of miR-101 enhances anti-tumor effect of TRAIL on PTC *in vivo* **(A)** Nude mice were subcutaneously inoculated with TPC-1/miR-101 cells followed by treating with 40 μg/kg TRAIL per two days. Tumors were purified and weighted after the mice were sacrificed. ^*^P<0.05 *vs.* TPC-1/control+TRAIL group. **(B)** Expression level of miR-101 in purified tumors was detected by qRT-PCR analysis. ^*^P<0.05 *vs.* TPC-1/control group. ^#^*P*<0.05 *vs.* TPC-1/control+TRAIL group. **(C)** Western blot analysis was performed to detect the protein levels of c-met and MCL-1, and phosphorylation of PI3K, AKT and Bad in purified tumors.

To investigate whether miR-101 enhances anti-tumor effect of TRAIL on PTC *in vivo*, we established a TPC-1 xenograft model by subcutaneously inoculating with TPC-1 cells which were transfected with lentivirus carrying miR-101 precursor sequence on nude mice. The results of *in vivo* experiments showed that sizes and weight of TPC-1 xenografts in TRAIL plus miR-101 group were obviously smaller than these in TRAIL single treatment group (Figure 6A). We therefore demonstrated that miR-101-overexpressed PTC cells were significantly more sensitive to TRAIL treatment *in vivo*. Subsequently, we measured the expression of miR-101 and c-met/MCL-1 in purified tumor tissues. We confirmed the overexpression of miR-101 in lentivirus-miR-101-transfected tumors (Figure 6B). Furthermore, the miR-101-overexpressed PTC tumors showed obviously lower protein level of c-met and MCL-1, and lower phosphorylation of PI3K, AKT and Bad (Figure 6C). Token together, we demonstrated that miR-101 decreased the expression of c-met and MCL-1 in PTC both *in vitro* and *in vivo*. Overexpression of miR-101 was able to enhance the anti-tumor effect of TRAIL on PTC.

## DISCUSSION

Drug resistance is a major obstacle for conservative treatment of PTC [26]. Although TRAIL has been found to exhibit anti-tumor effect on PTC, strategies are still required to increase the sensitivity of PTC cells to TRAIL. Recent studies have reported that certain miRNAs regulate TRAIL sensitivity in some cancers [19, 20]. However, relationship between miRNAs and TRAIL sensitivity in PTC is still unclear. Among these drug-related miRNAs, miR-101 is usually downregulated in cancers. Furthermore, miR-101 is found to sensitize gastric cancer, prostate cancer, bladder cancer and hepatocellular carcinoma cells to anti-tumor drugs [27, 28, 29, 30]. These reports demonstrate that miR-101 is a potential sensitizer for anti-tumor treatment. In the present study, we observed significant decrease of miR-101 expression in PTC cells compared to the human normal thyroid epithelial cells. Moreover, evidence was shown that miR-101 was associated with TRAIL sensitivity to PTC. We concluded that overexpression of miR-101 was able to enhance the anti-tumor effect of TRAIL on PTC both *in vitro* and *in vivo*.

Hepatocyte growth factor receptor (c-met), also called tyrosine-protein kinase MET, is an oncogene for promoting the tumorigenicity [31]. Abnormal c-met activation is responsible for poor prognosis in cancer patients [32], because activated c-met promotes tumor proliferation, metastasis and formation of new blood vessels [33, 34, 35]. Moreover, reports have proved that overexpression of c-met is correlated with failure of treatment with anti-tumor agents. Among the downstream of c-met pathway, phosphorylation of PI3K and the subsequent activation of AKT represents one key mechanism for formation of anti-apoptosis and drug-resistance [36, 37]. Therefore, c-met has been regarded as a promising target for cancer therapy.

Myeloid cell leukemia 1 (MCL-1) is a mitochondria-located anti-apoptotic protein. It belongs to Bcl-2 family and contains three Bcl-2 homology (BH) domains. MCL-1 can bind to the pro-apoptotic proteins such as Noxa, Puma and Bax, and then inactivate them [38, 39]. Therefore, MCL-1 is an important negative regulator for mitochondrial apoptosis. Indeed, overexpression of MCL-1 is revealed to prevent drug-induced apoptotic cell death in multiple cancers. Moreover, knockdown of MCL-1 has been considered as a novel strategy to enhance the effect of anti-tumor drugs including TRAIL [40, 41, 42, 43].

Recently, Studies have reported that both c-met and MCL-1 can be regulated by miRNAs, and the miRNAs-c-met/MCL-1 axis determined the drug sensitivity to several cancers [36, 21, 44, 45]. In this study, we observed significant overexpression of c-met and MCL-1 in PTC cells. We then showed that expression levels of c-met and MCL-1 were negatively correlated with TRAIL sensitivity in PTC cells. Interestingly, we found that both c-met and MCL-1 can be targeted by miR-101. Restored expression of miR-101 suppressed the functions of MCL-1 and c-met, and thus promoting mitochondrial apoptosis induced by TRAIL.

**Figure 7:**
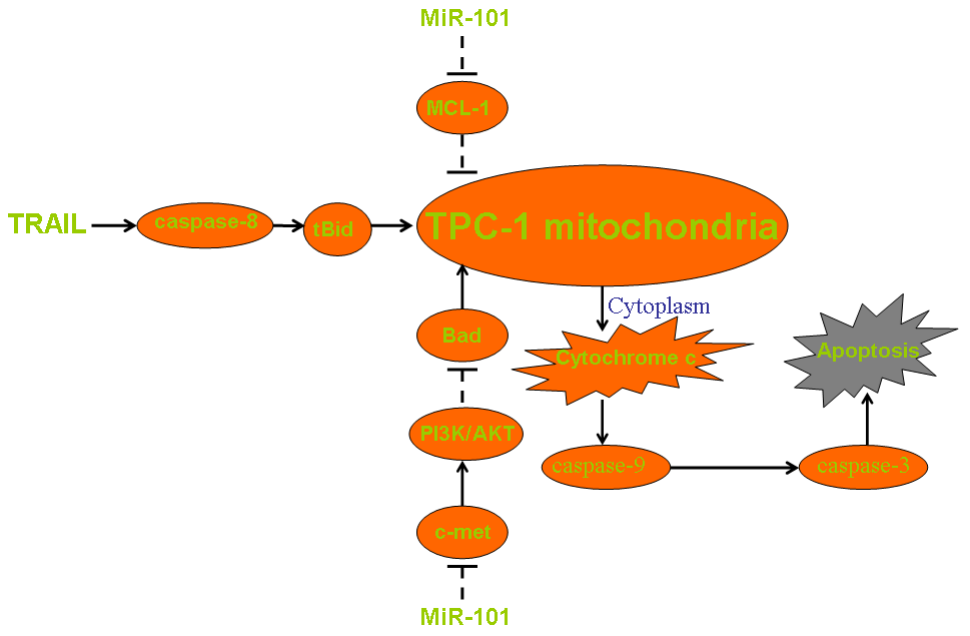
Pathway of mitochondrial apoptosis in miR-101 and TRAIL co-treated TPC-1 MiR-101 decreases the expression of c-met and thus inhibiting the phosphorylation of PI3K and AKT. Low phosphorylation level of AKT induces dephosphorylation of Bad and thus promoting collapse of mitochondria. Besides, miR-101 directly suppresses the expression of MCL-1 to impair the resistance of TPC-1 cells to apoptotic signals. These mechanisms facilitate TRAIL-induced damage of mitochondria, leading to the release of cytochrome c and the downstream of caspase-9 activation. Finally, cleaved caspase-9 triggers caspase-3 and the following apoptosis.

In summary, we showed the pathway of miR-101-c-met/MCL-1 axis on enhancing TRAIL-induced apoptosis in PTC (Figure 7). Although some PTC cells develop mechanisms against TRAIL-mediated apoptosis, our data provide a potential strategy to increase the sensitivity to TRAIL through introduction with some miRNA sensitizers such as miR-101.

## MATERIALS AND METHODS

### Cell culture

Human normal thyroid epithelial cell line Nthy-ori3-1 and the PTC cell lines TPC-1, BCPAP and K1 were purchased from the Institute of Biochemistry and Cell Biology, Chinese Academy of Sciences (Shanghai, China). Cells were cultured in Dulbecco’s modifed Eagle medium (DMEM, Gibco, Carlsbad, USA) containing with 10% fetal bovine serum (Gibco) at 37°C in a incubator added 5% CO_2_.

### Detection of miR-101 expression

Expression of miR-101 in PTC cell lines was detected by quantitative reverse transcriptase real time PCR (qRT-PCR) analysis. Briefly, total RNA was extracted from PTC cells by using the TRIzol reagent (Invitrogen, USA). Subsequently, miR-101 was reverse transcribed by using One Step PrimeScript miRNA cDNA Synthesis Kit (TaKaRa, Dalian, China) and quantified by using SYBR Premix Ex Taq (TaKaRa) under ABI PRISM 7900 Sequence Detection System (Applied Biosystems Prism, USA). Relative expression of miR-101 was normalized to U6 small nuclear RNA (snRNA) and determined by using the 2^-∆∆CT^ analysis method [46].

### Transfection

Control miRNA (miR-c), miR-101 mimics, miR-101 inhibitors (anti-miR-101), control siRNA (siRNA-c), c-met small interfering RNA (siRNA-c-met) and MCL-1 small interfering RNA (siRNA-MCL-1) were purchased from GenePharma Co. Ltd. (Shanghai, China). For enforced expression of c-met and MCL-1 in PTC, c-met and MCL-1 eukaryotic expression plasmid (plasmid-c-met and plasmid-MCL-1, respectively) was conducted by cloning the open reading frame of c-met or MCL-1 gene into the pcDNA3.1 plasmid (Life Technologies, USA). RNA oligonucleotides (50 pmol/ml) or plasmids (2 μg/ml) were transfected into PTC cells by using Lipofectamine 2000 (Invitrogen) according to the manufacturer’s instructions followed by incubating for 24 h.

### Cell viability assay

Transfected PTC cells were seeded into 96-well plates overnight before they were incubated with 2 ng/ml TRAIL for 48 h. Subsequently, MTT assays were performed to measure the cell viability as described previously [47]. The absorbance values of each well were determined at 570 nm by using a microplate reader (Sunrise Microplate Reader, TECAN, Switzerland). Relative cell viability in experimental groups was normalized to control group.

### Luciferase reporter assay

The wild and mutant 3’ UTR of c-met and MCL-1 genes were designed and prepared by GenePharma Co. Ltd. Subsequently, these prepared c-met and MCL-1 3’ UTR were cloned into the pGL3 Luciferase Reporter Vectors (Promega, USA). For luciferase reporter assay, TPC-1 cells were seeded into 48-well plates overnight, followed by co-transfection with miR-101 (50 pmol/ml), Renilla luciferase pRL-TK vector (100 ng/ml, Promega) and pGL3 plasmid with wild/mutant 3’ UTR of c-met/MCL-1 gene by using Lipofectamine 2000. 48 h post-transfection, luciferase assays were performed by using the Dual-Luciferase Reporter assay system (Promega) according to the manufacturer’s instructions. Renilla luciferase activities were normalized to firefly luciferase activities.

### Mitochondria removal

Before western blot analysis on cytochrome c, Mitochondria/Cytosol Fraction Kit (BioVision, USA) was used to separate the mitochondria and cytoplasm fractions in TPC-1 cells. We collected the cytoplasm fraction to detect the protein level of cytochrome c in following western blot assays.

### Immunoprecipitation

TPC-1 cells were lysed by using RIPA buffer on ice for 30 min followed by collection of the supernatants. Next, antibody of Bad (Cell Signaling Technologies, USA) was added into the supernatants and incubated overnight at 4 °C. Protein A agarose beads (Cell Signaling Technologies) were then added into the above supernatants and incubated for 2 h before centrifugation. Subsequently, precipitated agarose beads were washed with cold RIPA buffer, and the proteins binding to the agarose beads were then separated by boiling in sodium dodecyl sulfate (SDS) sample buffer and detected by western blot analysis.

### Western blot analysis

TPC-1 cells were lysed by using RIPA buffer on ice for 30 min followed by collection of the supernatants. Approximately 50 μg of total proteins in the collected supernatants were then separated by 10% SDS-PAGE followed by transfer to PVDF membranes (Millipore, USA). The membranes were blocked in 5% skim milk for 1 h at room temperature and then incubated with the following primary antibodies (Cell Signaling Technologies): anti-c-met, anti-MCL-1, anti-GPADH, anti-caspase-8, -9 and -3, anti-phosphorylated Bad, PI3K and AKT, anti-Bad, anti-PI3K, anti-AKT, anti-Bcl-xl and anti-cytochrome c. After incubation with primary antibodies overnight, the membranes were washed and incubated with appropriate horseradish peroxidase (HRP)-conjugated secondary antibody for 2h at room temperature. Proteins on the membranes were finally probed by using an enhanced chemilu-minescence detection kit (Pierce, USA).

### Detection of mitochondrial membrane potential (MMP) and cell apoptosis

5,5′,6,6′-Tetrachloro-1,1′,3,3′-tetraethyl imidacarbo cyanine iodide (JC-1, Molecular Probes, USA) and Annexin V-FITC Apoptosis Detection Kit (Sigma-Aldrich, USA) were used for detection of MMP and cell apoptosis respectively according to the manufacturer’s instructions. Both the MMP and cell apoptosis were analyzed by using a flow cytometry (Becton Dickinson, USA).

### *In vivo* tumorigenicity

Animal care and experimental protocols were approved by the Animal Care Committee of Linyi People’s Hospital. For preparation of *in vivo* experiments, Precusor sequence of miR-101 (5’-UGCCCUGGCUCAGUUAUCACAGUGCUGAUGCUGUCUAUUCUAAAGGUACAGUACUGUGAUAACUGAAGGAUGGCA-3’) was inserted into the lentivirus in Genechem Co., Ltd. 5×10^5^ transducing units of recombinant or empty lentivirus were then transfected into the TPC-1 cells. Subsequently, 5×10^6^ of transfected TPC-1 cells were inoculated subcutaneously into the 4-week-old, female nude mice (BALB/c, Shanghai Super-B&K Laboratory Animal Corp., Ltd., Shanghai, China). For *in vivo* treatment, TRAIL was administrated by intraperitoneal injection per two days (40 μg/kg). 31 days post-injection, mice were sacrificed before separating the tumors and weighting them. For detection of miR-101, c-met and MCL-1 expression in tumor tissues, collagenase type III was used to purify them.

### Statistical analysis

Experimental data were obtained from at least three times independently experiment. Quantitative data were expressed as the mean ± SD and analyzed by using SPSS 15.0 software. Differences between groups were compared by using Two-tail Student’s t test and ANOVA methods. *P*<0.05 was considered to be statistically significant.
